# Tonsillar Kaposi’s Sarcoma in HIV Positive Patient with Syphilis Infection

**DOI:** 10.22038/IJORL.2024.72743.3463

**Published:** 2024-03

**Authors:** Francesco Lorusso, Salvatore Alberto Di Vincenzo, Valerio Campofiorito, Federico Sireci, Angelo Immordino, Francesco Dispenza

**Affiliations:** 1 *Otorhinolaryngology Section, Department of Biomedicine, Neuroscience and Advanced Diagnostics, University of Palermo, AOUP “Paolo Giaccone”, Via del Vespro, 133, 90127 Palermo, Italy.*

**Keywords:** HIV, Kaposi’s sarcoma, Tonsillar tumor, Syphilis

## Abstract

**Introduction:**

Since the introduction of Highly Active Anti-Retroviral Therapy (HAART), there has been a significant increase in the survival of HIV-infected patients. Consequently, there has been increased attention on the potential neoplastic pathologies, such as Kaposi’s sarcoma, associated with AIDS in these individuals.

**Case Report:**

In this case report we present, for the first time, a patient affected by Kaposi's sarcoma of the palatine tonsil with a concomitant syphilis infection. The patient underwent enlarged tonsillectomy and continued antiretroviral therapy. There were no signs of disease recurrence at a 12-month follow-up.

**Conclusions:**

Despite the rarity of tonsillar localization of Kaposi's sarcoma, it should be suspected in the presence of an HIV-infected patient. Tonsillectomy effectively controls local disease, but comprehensive patient management requires a multidisciplinary team of healthcare professionals, including infectious disease specialists, pathologists, and oncologists who work together to provide high-quality and coordinated care.

## Introduction

Kaposi's sarcoma (KS), first described by the Hungarian dermatologist Moriz Kaposi in 1872, is a disease characterized by proliferative vascular lesions, almost all of which contain Kaposi's sarcoma-associated herpesvirus (KSHV), also known as human herpesvirus 8 (HHV 8). KSHV is an angiotrophic and lymphotropic herpesvirus, and its genome codes for several proteins involved in proliferation, angiotrophic functions, and inflammation (1). 

Approximately 25% of all human cancers are etiologically related to infections, including both bacterial and viral infections, which are generally controlled by the host immune system. 

However, in immunodeficient patients such as those with acquired immunodeficiency syndrome (AIDS) or patients undergoing immunosuppressive therapy for organ transplantation, the control over these infections is lost, leading to a significant increase in the incidence of cancers associated with infectious agents (2). 

Before the HIV/AIDS pandemic of the 1980s, which completely changed the patterns of KS, three forms of the disease were described: endemic, classic, and associated with transplantation immunosuppression (IT-KS) (3).

 Since the beginning of the HIV era, a new form of KS has emerged that exclusively affects people with HIV, known as AIDS-associated KS (AIDS-KS). While IT-KS tends to occur in the lower extremities, AIDS-KS primarily affects the head and neck region in about 70% of cases, and the oral cavity is involved in approximately one-third of the affected patients (1-3). 

Oral Kaposi's sarcoma may appear similar to gingival hyperplasia linked to cyclosporine in patients who have undergone organ transplants. However, cyclosporine generally leads to a widespread fibrotic enlargement of the gums, while oral Kaposi's sarcoma tends to cause more localized purplish-red swelling. (4). 

The hard palate, gingiva, and tongue are the most frequently affected sites in oral KS (5). Lesions can lead to tooth loss and are associated with pain, ulceration, and bleeding. Oropharyngeal KS, particularly isolated KS of the tonsil, is extremely rare.

## Case Report

**Fig 1:**
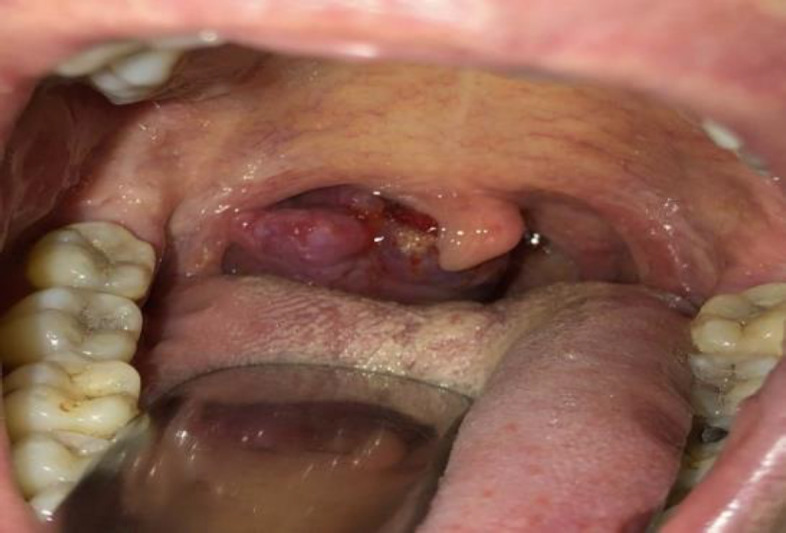
Pre-operative clinical examination: right tonsillar Kaposi’s sarcoma

A male patient affected by HIV and syphilis infection presented to our ENT department reporting pharyngodynia and dysphagia for about 2 weeks, along with occasional bleeding. The patient did not experience dyspnea or any other symptoms. Clinical examination showed a purplish swelling on the right palatine tonsil, with its largest diameter measuring about 2.5 cm. (Figure 1). 

**Fig 2:**
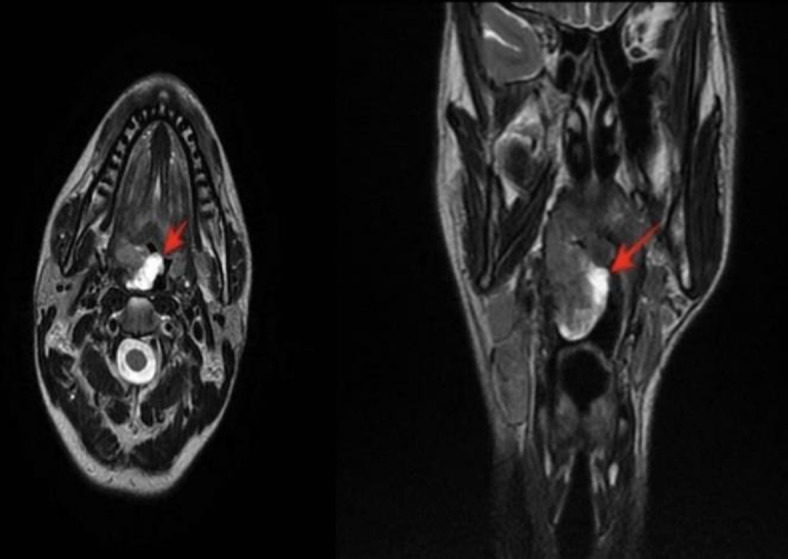
Head and neck contrasted MRI

The swelling crossed the midline, extending over the uvula. No other lesions were found in the oral cavity, pharynx, or larynx. Palpation of the neck revealed latero-cervical lymphadenopathy on the same side as the lesion. No KS skin lesions were detected. As the first step, a contrast MRI was performed, revealing a vascularized neoformation of the right palatine tonsil (Figure 2). 

**Fig 3:**
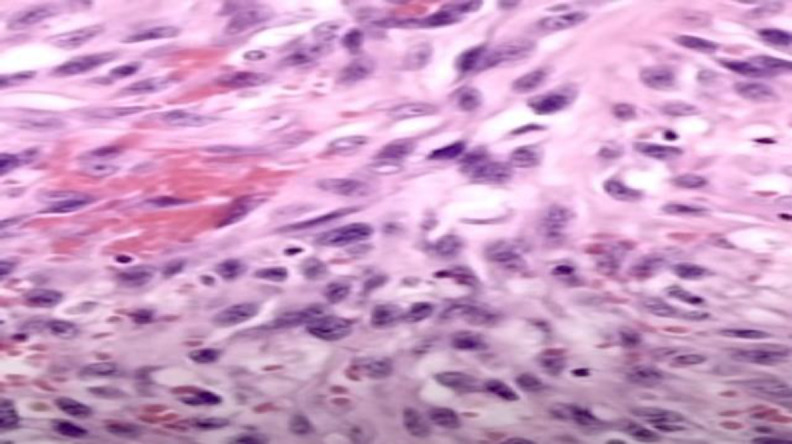
Histopathological features (Hematoxylin-eosin staining, 400x)

Subsequently, an incisional biopsy of the lesion was conducted, and the histopathological examination showed a nodular proliferation composed of monomorphic spindle cells arranged in fascicles, mixed with micro vessels associated with erythrocyte extravasation. Figure 3 shows the microscopic histopathological features observed with hematoxylin and eosin staining (Figure 3). 

**Fig 4:**
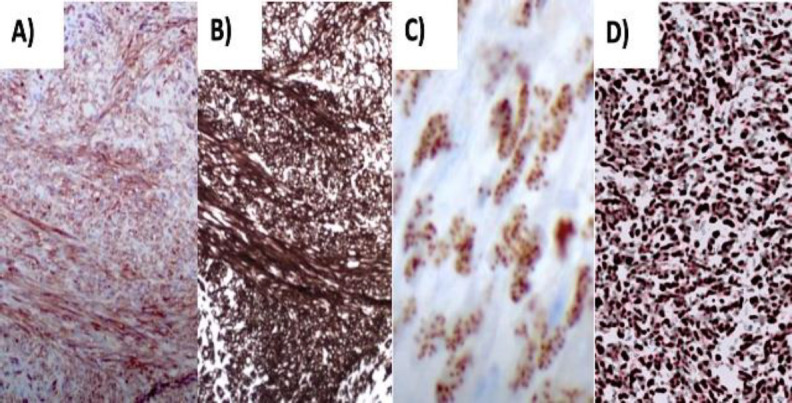
A) CD31 (100x), B) CD34 (100x), C) HHV8 (400x), D) MIB1 (200x)

The immunohistochemical examination revealed positivity for CD31, CD34 and HHV8, and a positivity for the MIB1 proliferation index of 5%. Figure 4 shows the microscopic immunohistochemical features (Figure 4). 

The morphological and immunophenotypic characteristics suggested a diagnosis of Kaposi's sarcoma. The treatment included surgical removal of the left tonsil, which confirmed the diagnosis of Kaposi's sarcoma. The tumor was completely excised through an enlarged tonsillectomy procedure (6-8). 

**Fig 5:**
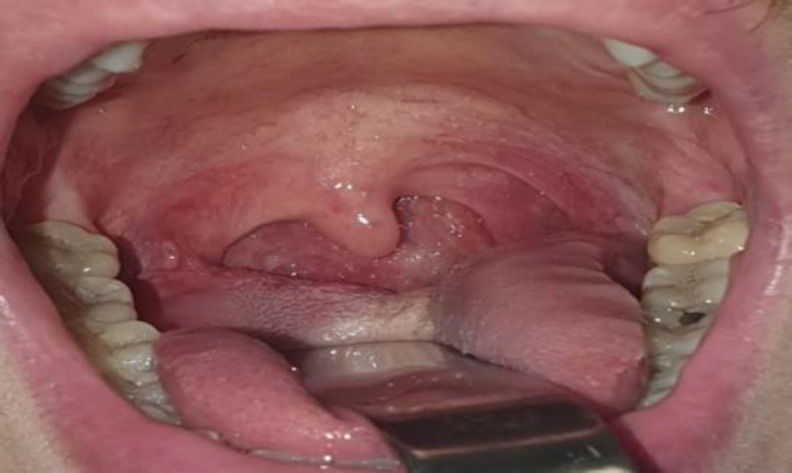
Post-operative clinical examination: no signs of recurrence after 12-months Follow-up

The patient had a regular post-operative course with complete recovery within ten days. The patient continued with antiretroviral treatment. Follow-up over a period of 12 months showed no signs of disease recurrence (Figure 5).

## Discussion

Kaposi's sarcoma is characterized as an angioproliferative disorder marked by lesions that display spindle cell growth, new blood vessel formation, inflammation, and edema (9,10). HHV8 has been identified in over 95% of all KS lesions, across all epidemiological subtype (11-13). 

The oncogenic mechanism of HHV8 has not been fully understood, as the virus is present in both immunosuppressed and immunocompetent individuals. However, HHV8 infection seems to play a major role in individuals with compromised immune status (14,15). Four clinical variants of KS with identical histological features but distinct epidemiological patterns have been identified: classic KS, endemic KS, transplant-related or immunosuppressed KS (IT-KS), and AIDS-associated KS (AIDS-KS). Since the introduction of HAART (highly active antiretroviral therapy) in high-income countries, a significant decrease in the incidence of AIDS-KS has been observed, with the estimated incidence being around 5%, according to the latest evidence (16-18). 

However, KS rates remain significantly higher in low- and middle-income countries. Compared to the classical and endemic forms of KS, IT-KS and AIDS-KS tend to be more aggressive. The clinical course of AIDS-KS can vary from an indolent, slowly progressive disease to a rapidly progressive and fatal course (19). AIDS-KS is associated with a shortened life expectancy, although most patients die from opportunistic infections or lymphoma, rather than KS itself (20). 

Oral KS is more likely to cause symptoms compared to cutaneous KS, which often leads to earlier detection by clinicians. The occurrence of KS in the tonsils often becomes noticeable to patients because of symptoms such as pharyngodynia and dysphagia. 

Moreover, the presence of ulcerated areas in the neoplasm can cause haemoptysis, leading patients to seek prompt medical attention, often in the Emergency Department. In such conditions, patients, who are probably already aware of their HIV status, are likely to share this information with their healthcare provider, facilitating the diagnosis of a suspected neoplasm related to AIDS. Once KS is suspected, confirmation is obtained through histopathological examination of biopsy specimens. The histopathology of different epidemiological types of KS is nearly identical, with minor differences observed between AIDS-KS and non-AIDS KS samples (21). 

Key features include angioproliferation, spindle cells, inflammatory infiltrate, and edema. Identification of HHV8 DNA can help distinguish KS from other vascular lesions (22). If KS is identified in a patient without a known history of HIV infection, an HIV test is strongly recommended. 

The evaluation of a patient with KS by an ENT specialist should be followed by a comprehensive examination of the skin and abdominal area to check for the presence of visceral lesions. Symptoms such as fever, night sweats, and weight loss should be noted, along with the patient's immunological status, including CD4 count and viral load. 

Tonsillar involvement is very rare, and only fourteen cases have been reported in the literature over the past 10 years (23-26). Furthermore, based on the available evidence, this represents the first case report of a patient affected by KS and syphilis infection. 

## Conclusions

Since the introduction of Highly Active Anti-Retroviral Therapy (HAART), there has been a significant increase in the survival of HIV-infected patients. Consequently, there has been increased attention on the neoplastic pathologies associated with AIDS in these individuals. These include Kaposi sarcoma (KS), non-Hodgkin's lymphoma, and invasive cervical cancer. These malignancies are known as AIDS-defining malignancies (ADMs) and their incidence rate appears to be significantly higher in the advanced stages of the disease. KS frequently appears in the oral cavity and often serves as the initial sign that leads to the disease’s diagnosis. However, tonsillar localization of KS is extremely rare compared to other sites in the oral cavity. In cases of suspected tonsillar KS, it is advisable to perform a biopsy to obtain histological confirmation and, if the patient does not report seropositivity for HIV, a test should be offered and performed. Furthermore, investigation of other body areas (such as the skin and abdominal viscera) should be done to check for possible secondary involvement. In our experience, tonsillectomy has been found to be an effective treatment for local disease control however, for proper patient management, a multidisciplinary team consisting of infectious disease specialists, pathologists, and oncologists is required.
